# Suicidal patients presenting to secondary and tertiary emergency departments and referral to a psychiatrist: a population-based descriptive study from Japan

**DOI:** 10.1186/s12888-018-1690-2

**Published:** 2018-04-25

**Authors:** Izumi Chihara, Ryusuke Ae, Yuka Kudo, Ritei Uehara, Nobuko Makino, Yuri Matsubara, Teppei Sasahara, Yasuko Aoyama, Kazuhiko Kotani, Yosikazu Nakamura

**Affiliations:** 1 Division of Public Health, Center for Community Medicine, Jichi Medical University, 3311-1 Yakushiji, Shimotsuke, Tochigi, 329-0498 Japan; 2 Department of Neuropsychiatry, Keio University School of Medicine, 35 Shinanomachi, Shinjuku-ku, Tokyo, 160-8582 Japan

**Keywords:** Psychiatry, Suicide, Suicide attempt, Emergency medical service, Emergency hospital, Referral, Suicide prevention, Japan

## Abstract

**Background:**

In Japan, although many suicidal studies were previously conducted in tertiary emergency department (ED) settings, no published studies have reported on suicidal patients presenting to the secondary EDs. The aim of the study was to describe the characteristics of suicidal patients and the referral rates to a psychiatrist overall and by type of facility.

**Methods:**

Questionnaires were sent to all secondary and tertiary EDs in Tochigi prefecture, Japan. Data were collected for cases who presented in September 2009. Chi-square, Fisher’s exact, and t-tests compared the results by gender and type of ED.

**Results:**

All 74 EDs responded to the survey. There were 81 patients who attempted or died by suicide (36 men and 45 women). The most common method of suicide attempt was drug overdose (57%) followed by stabbing (17%). About a half used prescription drugs to attempt or die by suicide. The majority had a history of psychiatric disorders, and 35% had previous suicide attempt. About a half were admitted to medical or surgical unit; 33% were discharged home; and 9% died. After excluding those who died, 53% were referred to a psychiatrist, but 47% were not referred to a psychiatrist. The referral rate was lower for cases seen at secondary EDs (38%) compared to tertiary EDs (67%).

**Conclusion:**

Although professional organizations suggest that suicidal patients are seen by a psychiatrist, many were not, especially at secondary EDs. Further research is needed to assure that suicidal patients presenting to EDs receive appropriate psychiatric assessment and follow-up after discharge.

**Electronic supplementary material:**

The online version of this article (10.1186/s12888-018-1690-2) contains supplementary material, which is available to authorized users.

## Background

In Japan, suicide is a major public health problem. The number of suicide deaths increased from 23,494 in 1997 to 31,755 in 1998, and remained high around 30,000 per year for about a decade [1]. More recently, mortality rate of suicide decreased from 23.4 per 100,000 population in 2010 to 18.5 per 100,000 population in 2015 [1], which may be attributed to measures taken by the Japanese government, local government, and private organizations to reduce suicide rates in Japan [2]. However, suicide remained to be the eighth leading cause of death of all ages, and it was among the top three leading causes of death among men and women aged 15 to 54 in 2015 [1].

A suicide attempt is one of the most important risk factors for future suicide [3–8]. Studies have shown that 40 to 60% of suicides have previous deliberate self-harm [3, 7–10]. Because medically serious or potentially lethal suicidal patients are likely to present to emergency departments (EDs) [11], and research has shown that about 16 to 24% of them will repeat the attempts with more lethal methods [12, 13], it is important to study the characteristics of suicidal patients presenting to EDs. EDs can serve as important sites of suicide prevention [14], and the information on who visits the ED for a suicide attempt would be needed to develop intervention strategies tailored toward them [15].

Previous studies on suicidal patients conducted in Japan have been clinical studies at a single medical institution, such as a university hospital or a community hospital [16–23]. All of these studies were conducted in tertiary ED settings; thus, research is limited regarding suicidal patients who visit the secondary EDs. Secondary EDs in Japan are generally small hospitals with 50—200 beds, likely to be understaffed, and likely be an institution without a psychiatric department. Secondary EDs are designed to provide care for moderately-acute patients, who may require inpatient care. Because emergency care system in Japan is not as centralized as in western countries, there are more small emergency hospitals (i.e., secondary EDs) in Japan than in western countries [24]. Several secondary EDs do not have specialists around the clock, and as a result, some have suggested that secondary EDs in Japan may lack the capacity to care for all types of emergencies [24, 25]. However, research is lacking regarding the current status of ED visits by suicidal patients and their characteristics in Japan.

Furthermore, research is limited with regard to how often the suicidal patients are referred to mental health professionals for psychiatric assessment in Japan despite the fact that professional organizations in Japan recommend or suggest that ED physicians refer suicidal patients presenting to EDs to psychiatric departments before patients are discharged home from the ED [26–28]. Suicide prevention opportunities may be missed if appropriate psychiatric assessments for suicidal patients are lacking in the EDs [29]. One study from the US has shown that 90% of pediatric patients who visited one of four EDs in Ohio for psychiatric-related illnesses had a mental health consult in the ED [30]. However, another study from the US reported that psychiatrists evaluate the majority of suicidal patients in only 10% of EDs in California [31]. The current situation has not been rigorously examined in Japan.

To our knowledge, there are no published studies that investigated the number and the characteristics of suicidal patients at secondary EDs in Japan, and how they may differ from patients presenting to tertiary EDs. There is also a gap in knowledge regarding referral rates to a psychiatrist in the EDs, and whether referral rates to psychiatric departments differ by type of facility (i.e., secondary vs. tertiary EDs).

Therefore, we conducted a population-based descriptive epidemiological study to investigate the characteristics of suicidal patients presenting to secondary and tertiary EDs in Tochigi prefecture, and to examine the referral rates to the psychiatric department overall and by type of facility. Specific purposes of the study were (1) to describe the characteristics of ED facilities that treat suicidal patients in Tochigi prefecture; (2) to describe the demographic, social, and clinical characteristics of suicidal patients (including attempted and completed suicide cases) presenting to secondary and tertiary EDs in Tochigi prefecture overall and by type of facility; and (3) to describe the rate of referral to a psychiatrist by ED physicians overall and by type of facility. Our main objective was to understand the population of suicidal patients presenting to EDs, and whether there may be missed opportunities at the EDs related to psychiatric referrals.

## Methods

### Data collection

Tochigi prefecture is located in Kanto Area, north of Tokyo, and has a population of about two million and an area of about 6408 km^2^. Tochigi is about mid-size in terms of population and area among 47 prefectures of Japan, and has experienced a higher suicide mortality rate (27.8 per 100,000 population; ranked 11th of 47 prefectures in 2009) compared to the national average (24.4 per 100,000 population) [1, 32]. There are 69 secondary EDs and 5 tertiary EDs in Tochigi prefecture.

A pen-and-pencil questionnaire survey was sent to all 74 secondary and tertiary EDs in Tochigi prefecture in August 2009, asking the directors, the administrative and/or clinical staff to collect information prospectively on suicidal patients (i.e., attempted suicide and suicide cases) that presented to the ED in the month of September 2009. Questionnaires asked about the characteristics of each institution as well as demographic, social, and clinical characteristics of all attempted suicide and suicide patients presenting to the ED for the study period, patient outcomes (e.g., death, discharged home), and services that they received. We asked that the clinical staff in charge of patient care fill out the part on each patient. A reminder to complete the survey was mailed in September and October. Questionnaires were sent again in October to institutions that had not returned the survey. All responses were collected via mail by the end of November.

### Definition of cases

This study used a broad definition of attempted suicide and suicide, with or without the intention to die [33]. The study aimed to capture all cases of attempted and completed suicide, regardless of suicidal intent, for the following three reasons. First, although we acknowledge that patients who engage in self-harm are a heterogeneous group [33], it is still important to describe this population as a whole since there is increased risk of later suicide among self-harm patients [34–37]. Second, it is difficult to differentiate between the different subgroups (e.g., those with and without intent), and the literature is divided on how to define subgroups of self-harm [33, 38, 39]. It would be even more challenging for the ED physicians to categorize cases into different subgroups of self-harm because they are not trained to do so. Finally, it is known that there is substantial overlap between suicides and serious suicide attempts [40]. Since cases presenting to secondary and tertiary EDs would be alive (at least at the time of presentation to ED) but will include medically serious cases [31], it is expected that there will be substantial overlap between attempted suicide (who present to the ED) and suicide cases (who may or may not present to the ED).

### Questionnaires

The questionnaires consisted of two parts, which are shown in Additional files 1 and 2. The first part asked about the participating medical institution, such as the number of beds, number of psychiatrists, and the number of total patients seen in the ED during the month of the study. The first part also asked about the number of attempted and completed suicide cases presenting to the ED during the month of September 2009. If there was any case identified by the administrative and/or clinical staff, they were asked to go on to the second part of the survey. The second part collected information on each suicidal patient (i.e., attempted or completed suicide case) that presented to the EDs during the same period, without reference to suicidal intention. The first part of the questionnaire was to be filled out by either administrative and/or clinical staff (either a physician or a nurse) while the second part was to be filled out by ED physicians in charge of patient care. The survey collected demographic and medical information of the suicidal patients as well as their outcomes and services provided.

### Data analysis

Descriptive analyses were conducted to describe the characteristics of EDs that treat suicidal patients. Descriptive analyses further described the demographic distributions of suicidal patients. Bivariate analyses, using Student’s t-test for numerical data and Chi-Square Tests or Fisher’s Exact Tests for categorical data as appropriate, were conducted to assess whether the characteristics of cases, patient outcomes, and referral rates differed by gender or by the type of institution (secondary vs. tertiary). Complete case analysis was used when data were missing on a variable. An alpha level of 0.05 was used to determine statistical significance. Due to small sample size, *p* values of less than 0.20 are also reported. All data management and analyses were conducted using Statistical Analysis System® (SAS, version 9.3; SAS Institute, Cary, NC). This study was approved by the Ethical Committee on Epidemiologic Research at Jichi Medical University (July 21, 2009, Eki09–09).

## Results

All 74 EDs in Tochigi prefecture responded to the survey (100% response rate). Overall, nine EDs (12%) had psychiatry departments in their facilities, of which, four EDs had on-site psychiatrists at night. There were a total of 51 full-time equivalent psychiatrists within all ED facilities. The total number of patients seen at all secondary and tertiary EDs in Tochigi was 19,939 in the month of September 2009.

Of all the 74 EDs, 19 facilities (26%) received at least one suicidal patient in the month of September 2009 (Table 1). A total of 81 suicidal patients were transferred to the 19 EDs. Of the 81 cases, 45 cases presented to one of five tertiary EDs while 36 cases presented to one of 14 secondary EDs. The rest of the 55 EDs (all were secondary EDs) reported that the number of suicidal patients was zero during the observed month. The average number of cases seen at the 19 facilities during September was 4.3. Overall, there were 4.1 attempted suicide/suicide cases per 1000 ED visits. There were 4.0 attempted suicide/suicide cases presenting to EDs per month per 100,000 population during the month of the study.

**Table 1:** Characteristics of 19 emergency departments (secondary vs. tertiary)

	Secondary EDs	Tertiary EDs	*P*-value ^a^
	(*n* = 14)	(*n* = 5)	
Mean number of beds in the facility (SD)	212 (176)	819 (302)	< .001
Mean number of emergency department visits (SD)	444 (371)	1589 (554)	< .001
Mean number of suicidal patients (SD)	2.6 (2.2)	9.0 (0.7)	< .001
Presence of psychiatrists in the facility			
At least one psychiatrist in the facility, n (%)	3 (21)	4 (80)	0.04
No psychiatrist in the facility, n (%)	11 (79)	1 (20)	
Mean number of psychiatrists in the facility (SD)	0.7 (1.7)	7.8 (9.5)	0.17

Emergency departments with at least one suicidal patient received in the month of September 2009 (of the total 74 facilities)

*Abbreviation*: *ED* emergency department, *SD* standard deviation

^a^Using Student’s t-test for numerical data and Fisher’s Exact Tests for categorical data, respectively

The demographic and clinical characteristics of suicidal patients (*N* = 81) are shown in Table 2. The average age of the cases was 46 years old, and the age ranged from 15 to 96. There was significant difference in age distribution between men (*n* = 36) and women (*n* = 45) (*p* = 0.003; Table 2). Men were older (average 55 years) compared to women (average 38 years). Majority (59%) of men were 50 years or older while 42% of women were in their teens or twenties. Overall, the majority were unemployed and resided with other family members. Over a quarter of cases had a previous suicide attempt as reported by the healthcare providers. More than half (60%) of them had the recent episode within a year (data not shown in table). Women were more likely than men to have a history of suicide attempt. There was no significant difference regarding sociodemographic and clinical characteristics between cases presenting to secondary or tertiary ED (Table 2).

**Table 2:** Sociodemographic and clinical characteristics of suicidal patients by gender, and by facility type (*N* = 81)

	Overall (*N* = 81)	Male (*n* = 36)	Female (*n* = 45)	*P*-value ^a^	Secondary EDs (*n* = 36)	Tertiary EDs (*n* = 45)	*P*-value ^a^
Gender, n (%)							NS
Male	36 (44)				18 (50)	18 (40)	
Female	45 (56)				18 (50)	27 (60)	
Age, years, n (%)				0.003			NS
15–29	22 (27)	3 (8)	19 (42)		12 (33)	10 (22)	
30–49	26 (32)	12 (33)	14 (31)		10 (28)	16 (36)	
50–69	17 (21)	10 (28)	7 (16)		8 (22)	9 (20)	
70+	16 (20)	11 (31)	5 (11)		6 (17)	10 (22)	
Occupational status, ^b^ n (%)				NS			NS
Unemployed	38 (67)	19 (68)	19 (66)		15 (68)	23 (66)	
In Occupation	19 (33)	9 (32)	10 (34)		7 (32)	12 (34)	
Residential status, ^b^ n (%)				NS			NS
Living with family	69 (90)	32 (94)	37 (86)		28 (88)	41 (91)	
Living alone	8 (10)	2 (6)	6 (14)		4 (13)	4 (9)	
History of previous suicide attempt, ^b^ n (%)				0.02			0.12
Yes	20 (35)	5 (19)	15 (48)		6 (24)	14 (43)	
No	37 (65)	21 (81)	16 (52)		19 (76)	18 (56)	
History of psychiatric disorder, ^b^ n (%)				0.06			NS
One or more psychiatric disorders	48 (71)	17 (59)	31 (79)		20 (77)	28 (67)	
None	20 (29)	12 (41)	8 (21)		6 (23)	14 (33)	

Percentages may not add up to 100% because of rounding

*Abbreviation*: *ED* emergency department, *NS* not significant

^a^Chi-Square Tests or Fisher’s Exact Tests ^b^Analyzed excluding unknown status: Occupational status (unknown *n* = 24), Residential status (unknown *n* = 5), History of previous suicide attempt (unknown *n* = 24), and History of psychiatric disorder (unknown *n* = 13), respectively

Most suicidal patients (*n* = 48) had a history of psychiatric disorders in the past. Overall, 38% of the cases either had a history of depressive disorders or had seen a psychiatrist due to depressive symptoms in the past (Table 3). There were no differences in the distributions of past psychiatric illnesses by gender or by type of institution (secondary vs. tertiary ED; data not shown).

**Table 3:** Prevalence of previously diagnosed psychiatric disorders among suicidal patients (*N* = 81)

Diagnosis	n (% of all patients)
Depressive disorder and/or depressive symptoms	31 (38)
Bipolar disorder	2 (2)
Schizophrenia	2 (2)
Anxiety disorder	2 (2)
Eating disorder	2 (2)
Personality disorder	1 (1)
Development disorder	1 (1)
Alcoholic disorder	1 (1)
Epilepsy	1 (1)
Diagnosis unknown	9 (11)

A total of 48 patients had previously diagnosed psychiatric disorder(s)

Some patients had more than one diagnoses: One patient had depressive disorder and/or depressive symptoms and epilepsy; one patient had depressive disorder and/or depressive symptoms and personality disorder; one patient had depressive disorder and/or depressive symptoms and eating disorder; and one patient had bipolar disorder and alcoholic disorder

There were three national holidays and four weekends (four Saturdays and four Sundays) in the month of September 2009 in Japan. For both men and women, the average numbers of cases were the highest for Mondays and the days after a holiday (Table 4). Men were more likely to attempt/die by suicide on weekdays than on weekends/holidays while women were more likely to attempt/die by suicide on holidays or weekends, although this difference by gender was not statistically significant (*p* = 0.11; Table 4).

**Table 4:** Average number of suicidal patients by day of week and holiday (*N* = 81)

	Average number of cases per day			*p*-value^a^
	Overall	Male	Female	
Day of the week				0.02
Monday	4.0	1.8	2.3	
Tuesday	2.8	1.2	1.6	
Wednesday	2.8	1.6	1.2	
Thursday	1.5	1.0	0.5	
Friday	2.0	0.8	1.3	
Saturday	2.5	0.8	1.8	
Sunday	2.8	1.0	1.8	
Holiday				<.001
Holidays or weekends	2.5	0.8	1.7	
Weekdays	2.7	1.4	1.3	
Day before or after a holiday				
Day before a holiday	2.0	0.8	1.3	NS
Day after a holiday	4.3	2.3	2.0	NS

Two patients were missing information on the day they attempted/died by suicide

^a^Chi-Square Tests, weighted by number of days in each category

The most common method of suicide attempt was drug overdose (57%) followed by stabbing (17%), hanging (7%), and jumping from height (7%; Table 5). A half (49%) used prescription drugs to attempt suicide. Many of them (42% of total) used minor tranquilizers, of which, all were benzodiazepines. Antidepressants were used in 10 cases (12% of total). Methods of attempted suicide/suicide and types of drugs used to attempt or die by suicide did not differ by gender or by type of institution (data not shown).

**Table 5:** Methods of suicide and drug types used to attempt or die by suicide (*N* = 81)

	n (% of all patients)
Method of Suicide (not mutually exclusive categories)^a^	
Drug overdose	46 (57)
Stabbing	14 (17)
Hanging	6 (7)
Jumping from height	6 (7)
Pesticide poisoning	5 (6)
Immolation	3 (4)
Carbon monoxide poisoning	2 (3)
Other	1 (1)
Type of drug used to attempt or die by suicide	
Prescription drugs only	39 (48)
Over the counter drugs only	6 (7)
Both prescription and over the counter drugs	1 (1)
Type of prescription drugs used to attempt or die by suicide (not mutually exclusive categories)^b^	
Minor tranquilizer	34 (42)
Major tranquilizer	7 (9)
Other psychoactive drug	15 (19)
Other drug	10 (12)

Methods used by patients who died by suicide (*n* = 7) included: hanging (*n* = 2), immolation (*n* = 2), pesticide poisoning (*n* = 2), and drug overdose (*n* = 1)

^a^Two patients used two methods (both drug overdose and stabbing)

^b^Of the 40 patients who used prescription drugs to attempt suicide, 31 patients used a combination of prescription drug types listed

The outcomes of suicidal patients and referral rates to a psychiatrist are shown in Table 6. About a half was admitted to medical or surgical service; 6% were admitted to psychiatric departments; and 33% were discharged home. After excluding seven suicide cases who died and one case missing information on referral, overall referral rate to a psychiatrist was 53%. Overall referral rate was significantly lower among patients at secondary EDs compared to tertiary EDs (38% vs. 67%, respectively; *p* = 0.02). Of the cases that were not referred to a psychiatrist (*n* = 34), the ED physicians felt the need to refer the patient to a psychiatrist 53% of the time (*n* = 18; data not shown in table). The main reasons why those patients were not referred to a psychiatrist were because patient or family refused it (33%), psychiatrists were unavailable (28%), or the patient was in coma (22%; data now shown in table). Referral rate was much lower for patients who visited facilities without a psychiatrist compared to patients who visited facilities with a psychiatrist (28% vs. 70%, respectively; *p* < .001). Most of the facilities without a psychiatrist were secondary EDs (Table 1). The outcomes of patients or referral rates did not differ significantly by gender (data not shown).

**Table 6:** Outcome of suicidal patients and referral to a psychiatrist (*N* = 81)

	Overall	Secondary EDs	Tertiary EDs	*P*-value ^a^
	n (%)	n (%)	n (%)	
Outcome of suicidal patients (*n* = 81)	[*n* = 81]	[*n* = 36]	[*n* = 45]	
Discharged home	27 (33)	12 (33)	15 (33)	NS
Died	7 (9)	1 (3)	6 (13)	0.09
Admitted to medical/surgery department of the institution	38 (47)	18 (50)	20 (44)	NS
Admitted to medical/surgery department of other institution	3 (4)	2 (6)	1 (2)	NS
Admitted to psychiatric department of the institution	3 (4)	1 (3)	2 (4)	NS
Admitted to psychiatric department of other institution	3 (4)	2 (6)	1 (2)	NS
Any referral to a psychiatrist (*n* = 73) ^b^	[*n* = 73]	[*n* = 34]	[*n* = 39]	0.02
Any referral to a psychiatrist	39 (53)	13 (38)	26 (67)	
Not referred to a psychiatrist	34 (47)	21 (62)	13 (33)	
Referral to a psychiatrist (*n* = 73) ^b^				0.03
Confirmed to have seen a psychiatrist	29 (40)	8 (24)	21 (54)	
Referred but not confirmed if the patient saw a psychiatrist	10 (14)	5 (15)	5 (13)	
Not referred to a psychiatrist	34 (47)	21 (61)	13 (33)	

Percentages may not add up to 100% because of rounding

*Abbreviation*: *ED* emergency department, *NS* not significant

^a^Chi-Square Tests or Fisher’s Exact Tests

^b^Excludes seven cases who died by suicide and one case missing information on referral

## Discussion

To our knowledge, this was the first published population-based study that examined the characteristics of suicidal patients presenting to secondary and tertiary EDs and that reported the referral rates to a psychiatrist overall and by type of facility. Despite the fact that guidelines recommend ED clinicians to consult the suicidal patients to a psychiatrist [26–28], in our study, 47% of those alive were not referred to a psychiatrist. Of the cases that were not referred to a psychiatrist, the ED physicians felt the need to refer the patient to a psychiatrist 53% of the time. Referral rate was especially low for cases seen at secondary EDs even though characteristics of suicidal patients were similar between secondary and tertiary EDs. These results are concerning because psychiatrists play a major role in treating the mental illnesses that suicidal patients may have in Japan [41]. Appropriate treatment of medical conditions (especially depression) is associated with reduced likelihood of suicide [42]. If suicidal patients are never referred to a psychiatrist or other mental health professionals after a suicide attempt, opportunities for suicide prevention may be missed [29].

In our study, the top reason for not referring the patient to a psychiatrist was patient and/or family refusal. The reasons for patient and family refusal were not studied, but one possible explanation is the stigma associated with mental health conditions and psychiatric treatment in Japan [43]. Another possible explanation is lack of knowledge about treatment effectiveness among patients and families. One study has shown that the general public in Japan lacks the knowledge that treatments are effective for mental health conditions such as schizophrenia and depression [44]. The same study also showed that the general public in Japan has a strong belief in counsellors, but a psychiatrist was not typically thought as most helpful for mental illnesses. Further research is needed to examine how stigma, knowledge, and beliefs around mental health diagnosis, treatment, and mental health professionals affect patient and family behaviors.

Another major reason why the patients were not referred to a psychiatrist was unavailability of a psychiatrist. There is chronic shortage of psychiatrists in Japan. In 2010, it was estimated that there were 10,844 full-time equivalent psychiatrists in Japan, but the Japanese Ministry of Health, Labour, and Welfare estimated that the number needed to increase to 12,045 to meet the needs [45]. In 2010, for Tochigi prefecture, there were 191 full-time equivalent psychiatrists while the estimated number needed was 207 [45]. The current study showed that there were only a total of 51 full-time equivalent psychiatrists within all the secondary and tertiary emergency hospitals in Tochigi prefecture in 2009, which cover about two million population. Japanese Ministry of Health, Labour, and Welfare does not estimate the number of psychiatrists that need to be available at secondary and/or tertiary EDs, but this number also likely needs to be increased. Nonetheless, there were a total of 191 psychiatrists in Tochigi prefecture at the time of the study [45]. The fact that referral rate was lower at facilities without a psychiatrist (mostly secondary) compared to facilities with a psychiatrist (28% vs. 70%, respectively) indicate that there may be a linkage problem between the EDs and psychiatrists in the community. Psychiatrists who do not work at the facilities with an ED may work at facilities without an ED, stand-alone clinics or stand-alone psychiatric hospitals. The possibility of linking EDs and psychiatrists in the community needs to be explored.

Basic demographic features in our study were similar to those reported in an unpublished study by Tokyo Metropolitan Government involving 422 suicidal patients presenting to 199 EDs in Tokyo Metropolis in the month of December, 2007 [32, 46]. There were 4.1 suicidal patients per 1000 ED visits in Tochigi, which are equivalent to the rates reported in Tokyo Metropolis. In our study, the in-hospital mortality rate was 9% among suicidal patients presenting to secondary/tertiary EDs while this rate was 12% for Tokyo Metropolis [46]. Another study by Ichimura et al. [16] also showed a similar in-hospital mortality rate of 9% among suicidal patients presenting to a single tertiary ED in Japan. Although large scale national studies on suicidal patients presenting to EDs in Japan have not been conducted, our data are consistent with past research in Japan and are likely representative of the situation in Japan.

In our study, at least a quarter of cases had a history of previous suicide attempt, and 60% of them had a recent episode within a year, according to the healthcare providers who completed the survey. These numbers may be an underestimate since the information was not collected directly from the patients. Studies on completed suicides in Japan using police data have shown that 13.4% to 15.9% of suicide cases had previous suicide attempt [4, 32]. According to a study by Inoue et al. [4], visiting a physician for an outpatient treatment was associated with previous suicide attempts in suicide cases. These results stress the importance of referring suicidal patients to proper healthcare when they are presented to EDs. Our study also showed that the majority (71%) had seen a psychiatrist for a psychiatric disorder. Medical professionals, including primary care physicians, ED physicians, and psychiatrists are regarded as one of the key players in preventing future suicides among suicide attempters [41], because medical professionals encounter these patients multiple times as shown in this study and prior studies. Suicide is a preventable event [47], and these results suggest that there clearly are opportunities for intervention by medical professionals, including ED physicians, primary care physicians, and psychiatrists [47]. The types of interventions that should occur between suicidal patients and medical professionals may be difficult to assess because treatment and follow-up need to be tailored to the broad needs of individual patients, most of whom have complex mental illness [47]. Nonetheless, this is the current gap in the literature that needs to be filled by future studies. For example, the interventions at the psychiatric visits such as counseling and medications must be re-evaluated since the visit itself has not been shown to be effective in preventing suicide attempt in this population.

Our study showed that 90% of cases were living with a family member(s). A study by Inoue et al. [4] also showed that living with a family member was associated with previous suicide attempts in suicide cases. It has been reported that conflicts among family members was the third common reason for suicide in Tochigi [32]. Further study is needed to examine if and how family factors can be a risk or protective factors for suicide attempt in Japan.

While suicide rate is higher for men, presentation of suicidal patients at healthcare facilities has been reported to be more common in women in Western countries [48]. Similar trends have been observed in Japan. In 2010, suicide rate for men was 2.6 times greater than that for women (34.1 per 100,000 for men and 13.1 per 100,000 for women) [1]. In our study, the proportion of female cases presenting to EDs was 56%, which is equivalent to what has been reported in Japan (between 56% and 63%) [17–19]. However, our study also showed that age distribution of men presenting to EDs is quite different from that of Western countries. While WHO/EURO study showed that men aged 25 to 34 and women aged 14 to 24 comprised the highest proportion of suicidal patients presenting to hospitals [48], our study showed that middle-aged and elderly men as well as young women (aged 15 to 29 years) comprised the highest proportions. A study from Akita prefecture among patients who present to healthcare facilities showed that attempted suicide was prevalent among men in their 20s and men aged 40 to 59 years while suicide death was most prevalent among men aged 40 to 69 years [49]. Although some claim that completed and attempted suicide population may be different in nature [40, 49, 50], there may be some overlap especially among men because the age distribution between attempted suicide cases and suicide cases overlap. These issues need to be examined in future studies.

In our study, the average numbers of cases were the highest on Mondays and the day after a holiday(s). Men had the highest number of cases earlier in the week (Mondays, Tuesdays, and Wednesdays). These findings are similar to the studies of completed suicide cases reported in Japan [32, 51]. A study by Nakamura et al. [32] on all cases of completed suicides in Tochigi prefecture in 2007 and 2008 also showed that the number of completed suicide cases were the highest on Mondays, Tuesdays, and Wednesdays. Furthermore, a study by Nishi et al. [51] investigated the relationship between suicide and holidays using vital statistics, and found that the highest rate of suicide was found for Mondays and the day after a holiday(s) for both men and women. Our results suggest that attempted suicide and suicide cases presenting to EDs share the similar traits to completed suicide cases (who may or may not present to the ED) in terms of the day of the week they tend to die by suicide. Mondays and the days after a holiday are when most people go back to work or school after a holiday. The people at high risk for suicide should not be left alone on those days. Private community organizations and local government may be able to make arrangements to visit previous suicidal patients on such days [41]. In addition, it is important to educate the family members and the general public on the risk of suicide and suicide attempt on Mondays, on the days after a holiday, and earlier in the week in general.

In our study, the majority of cases had a history of one or more psychiatric disorders. A total of 38% of cases had depressive disorders or had seen a psychiatrist for depressive symptoms. We did not investigate whether these patients were prescribed antidepressants, but only 12% of them used antidepressants to attempt or die by suicide while 42% used benzodiazepines to attempt or die by suicide. It is known that psychiatric disorders are present in the majority of suicide cases, and most of them are undertreated [52, 53]. Depression is undertreated even among patients who previously attempted suicide [54]. Suicidal patients need be referred to psychiatrists in order to receive appropriate psychiatric assessment for mood disorders so that they be diagnosed and be treated with antidepressants appropriately. This may decrease future suicide rates [55, 56]. On the other hand, appropriate use of benzodiazepines is in question because 42% of the cases used benzodiazepines to attempt or die by suicide. A study by Fukuhara et al. [57] showed that internists in Japan overprescribe benzodiazepines to patients with depressive symptoms without the use of antidepressants, which may increase the risk of future suicide and mortality. Benzodiazepines are prescribed more in Japan than anywhere else in the world [58]. Studies have shown that psychiatrists are 1.5 to 2 times more likely to prescribe benzodiazepines in Japan although the appropriateness of prescription by specialty is unknown [59]. Future studies need to examine if patients are prescribed benzodiazepines appropriately in Japan, and whether these patients may benefit from taking antidepressants or receiving (additional) psychotherapy instead.

There are some limitations in our study. Data on suicidal patients were collected only for the month of September. As a result, the sample size was small, and there was inadequate power to conduct multivariable analyses or show statistical significance in sub-analyses. In addition, we were not able to investigate the seasonal variation that may have occurred over the course of a year, which is known to occur among the suicidal population [51, 60]. We had to limit the data collection period to one month because Tochigi has several medically underserved areas. We felt that the study could not be conducted for a longer period of time due to the burden on ED physicians in Tochigi. EDs in Tochigi are already overcrowded, and ED physicians are overworked due to physician shortage [45], which is experienced in various parts of Japan. Nonetheless, we successfully investigated the state of the problem at current EDs.

Another limitation is that we relied solely on reports by administrative and/or clinical staff of each ED about information on cases, which may not have been accurate. Since we did not verify the numbers of attempted suicide and suicide cases by chart review, there is a possibility of underreporting of cases, especially at secondary EDs, where they may be understaffed to collect the information prospectively. Another source of bias associated with the data collection method is that, although this study used a broad definition of attempted suicide and suicide with or without the intention to die, it is possible that ED physicians did not report the cases involving less lethal methods, which will result in over-representation of severe cases in our sample. These biases may have affected our results, but the extent of bias is unknown.

In addition, because the questionnaires on each case were filled out exclusively by ED clinicians, no information was obtained directly from the patient, family members, or psychiatrists. Reports on psychiatric diagnosis by ED physicians may not be accurate especially if the patient had been diagnosed at a psychiatric department of another institution. No detailed information about the patient history related to mental illness (e.g., treatment received) was available. Personal information of the patient such as occupational status may not be accurate as well. Nevertheless, the strengths of the study are that it is population-based, covering both secondary and tertiary EDs, and the 100% response rate from eligible EDs.

In Japan, Basic Law on Suicide Countermeasures, which was passed into law in 2006 and revised by the Diet in 2016, specifically states that national government and local public entities should implement necessary policies to secure “effective coordination among psychiatrists and medical doctors who provide emergency medical care” [61]. Low referral rates to a psychiatrist especially at secondary EDs demonstrated in this study suggests that service coordination between secondary EDs and psychiatric departments/hospitals may be a pressing issue. Secondary EDs often do not have psychiatric departments in their facility as shown in this study, and they may not have the resources to link the patients to psychiatric services. Local governments should take initiatives in connecting the EDs to psychiatric hospitals and community resources. Because the number of psychiatrists is limited and not likely to increase soon, the role of other mental health professionals such as nurses, psychologists, and social workers in following up these patients needs to be examined. Recently, a multicenter randomized controlled trial examined the feasibility and effectiveness of assertive case management at 17 hospital EDs in Japan [62]. The study showed that assertive case management reduced the incidence of recurrent suicide attempt up to 6 months, but long term effectiveness was not shown. Similarly, a meta-analysis showed that active contact and follow-up type interventions for suicidal patients who visit EDs were effective for 12 months, but not at 24 months [63]. Nonetheless, these studies are promising that adverse outcomes could be delayed for 6 to 12 months. This is very relevant for clinical practice in Japan since this study showed that more than half (60%) of suicidal patients who previously attempted suicide had the recent episode within a year. Additional interventions are likely needed to improve long-term outcomes beyond 12 months for these patients.

Because this study only investigated the referral rates to a psychiatrist, future studies need to investigate whether suicidal patients presenting to EDs actually receive appropriate psychiatric assessment/evaluation and follow-up after discharge, and whether long term outcomes can be improved (e.g., recurrent suicide attempt and suicide mortality). Furthermore, studies need to examine further what components of interventions are effective in preventing future suicides in the ED settings and in psychiatric settings, taking into consideration the real-world situation and cultural context of Japan.

## Conclusions

The study investigated the characteristics and outcomes of suicidal patients presenting to secondary and tertiary EDs in Japan, and the referral rates to a psychiatrist. Findings from our study suggest that psychiatric referral rates were low among suicidal patients presenting to secondary and tertiary EDs in Japan. The study also showed that referral rates were lower in secondary EDs compared to tertiary EDs even though the characteristics of suicidal patients presenting to secondary and tertiary EDs were similar. Future studies should examine the outcomes of patients who are not referred to a psychiatrist at the EDs, and should examine what components of interventions are effective in preventing future suicides among suicidal patients who present to EDs.

## Additional files

Additional file 1: Questionnaire 1. Survey of Suicidal Patients in Tochigi Prefecture Questionnaire about Facility. (PDF 114 kb)

Additional file 2: Questionnaire 2. Survey of Suicidal Patients in Tochigi Prefecture Questionnaire about Suicidal Patients (Including Completed Suicide). (PDF 105 kb)

## Abbreviations

ED: Emergency departments
