# Supplementary material for: Suicidal patients presenting to secondary and tertiary emergency departments and referral to a psychiatrist: a population-based descriptive study from Japan
Source: BMC Psychiatry. 2018 Apr 25;18:112. doi: 10.1186/s12888-018-1690-2 (PMC5921746; doi:10.1186/s12888-018-1690-2)
Supplement: Supplementary file 1 — Questionnaire 1. Survey of Suicidal Patients in Tochigi Prefecture Questionnaire about Facility. (PDF 114 kb) [file 12888_2018_1690_MOESM1_ESM.pdf]

Survey of Suicidal Patients in Tochigi Prefecture
Questionnaire about Facility

Name of Facility: ____

Person filling out the form

Name: ____

Title: ____

Occupation 1. Physician 2. Nurse 3. Administrative Staff
4. Other ________________________

Phone number: ________ — ________ — __________ (Extension : ________________)

Total number of beds in your facility: (__________) Beds

Does your facility have a psychiatric department? If so, please answer additional questions.
For part-time psychiatrists, please enter the full-time equivalent.

1. Yes 2. No psychiatric department
↓
Number of psychiatrists

Full time: (__________) persons
Part time: (__________) persons [Full time equivalent]

Number of psychiatric beds: (__________) beds

On call psychiatrist present: 1. Yes 2. No

Please select the type of ED facility: 1. Secondary 2. Tertiary

For the following questions, please enter the number of ED visits September 1st and September 30th, 2009.
If the same patient visited multiple times, please count them as multiple visits.

A total number of visits during the month of September 2009: (__________) visits

A total number of visits by suicidal patients: (__________) visits

For each visit by suicidal patients, please proceed to questionnaire about patients.

If you have comments or suggestions about suicide prevention or about coordination of care between emergency departments and psychiatric departments, please describe below.

Please return this this questionnaire by October 16th, 2009 using the enclosed envelope.
Even if you did not have a suicidal patient visit at your facility, please return the form.
Thank you for your participation.

Jichi University Department of Epidemiology
Yosikazu Nakamura, MD, PhD, MPH
