# Supplementary material for: Suicidal patients presenting to secondary and tertiary emergency departments and referral to a psychiatrist: a population-based descriptive study from Japan
Source: BMC Psychiatry. 2018 Apr 25;18:112. doi: 10.1186/s12888-018-1690-2 (PMC5921746; doi:10.1186/s12888-018-1690-2)
Supplement: Supplementary file 2 — Questionnaire 2. Survey of Suicidal Patients in Tochigi Prefecture Questionnaire about Suicidal Patients (Including Completed Suicide). (PDF 105 kb) [file 12888_2018_1690_MOESM2_ESM.pdf]

Survey of Suicidal Patients in Tochigi Prefecture

Questionnaire about Suicidal Patients (Including Completed Suicide)

For suicidal patients who visited the ER between September 1st and 30th, 2009, please enter the information about each patient visit.
If a patient visited multiple times, please enter the information for each visit.

Date of ED visit: September (______), 2009

Sex: 1. Male 2. Female

Age: (______) years
If exact age is unknown, estimated age (______)

Address: 1. Tochigi Prefecture 2. Outside of Tochigi Prefecture 3. Unknown

Occupation: 1. In Occupation/Training
(a. Self-employed b. Management position c. Employee d. Student e. homemaker)
2. Unemployed 3. Unknown

Residential status: 1. Living with family 2. Living alone 3. Unknown

Mode of transport: 1. Ambulance 2. Transfer from another facility
3. On foot 4. Other (________________________)

Date and time of suicide attempt: Month (__________) Date (______), 2009
Time AM PM (__________)
Unknown

Method of suicide: 1. Hanging 2. Jumping in front of a train 3. Jumping from height
4. Stabbing
a. neck b. wrist c. chest d. abdomen e. other (__________)
5. Drug overdose
a. prescription drugs --- specify (________________________)
b. non-prescription drugs --- specify (________________________)
6. Gas
a. hydrogen sulfide b. other --- specify (________________________)
7. Pesticide poisoning --- specify (________________________)
8. Other --- specify ________________________)
9. Unknown

Drug abuse (including prescription drugs, but not including alcohol):
1. Yes --- drug name (________________________)
2. No
3. Unknown

History of psychiatric illnesses: 1. Yes --- diagnosis (______________________________)
2. No
3. Unknown

Alcohol use at the time of visit: 1. Use alcohol 2. Did not use alcohol 3. Unknown

History of alcoholism: 1. Yes 2. No 3. Unknown

Outcome:
1. Died
2. Admitted to psychiatric department of the institution
3. Admitted to other department of the institution --- department (________________)
4. Transferred and admitted to psychiatric department of other institution
5. Transferred and admitted to other department of other institution
--- department (__________________)
6. Discharged home
7. Other --- specify (____)

Referral to a psychiatrist (Select all the apply. If none applies, select 10.):

1. Admitted to psychiatric department of the institution
2. Admitted to psychiatric department of other institution
3. Transferred to other institution that has a psychiatric department
4. Evaluation by a psychiatrist of the institution
5. Evaluation by a psychiatrist of other institution
6. Referral paper to a psychiatrist was provided to the patient
(confirmed to have seen a psychiatrist)
7. Referral paper to a psychiatrist was provided to the patient
(not confirmed to have seen a psychiatrist)
8. Consultation to a psychiatrist via phone
9. Other involvement by a psychiatrist --- explain (______________________________)
10. No involvement by a psychiatrist

→ Did you feel a psychiatrist was needed?
1. No
2. Yes, but unable --- Reason (______________________________________)

Does this patient have a history of attempted suicide?

1. Yes --- Most recent suicide attempt: (______) days/months/years ago
2. No
3. Unknown

Is this patient a wrist cutter?

1. Yes
2. No
3. Unknown

Thank you for your participation.
Please return this form by October 16th, 2009 using the enclosed envelope.

Jichi Medical University Department of Epidemiology
Yosikazu Nakamura, MD, PhD, MPH
